# Error in the Sampling Area of an Optical Disdrometer: Consequences in Computing Rain Variables

**DOI:** 10.1155/2013/369450

**Published:** 2013-06-09

**Authors:** R. Fraile, A. Castro, M. Fernández-Raga, C. Palencia, A. I. Calvo

**Affiliations:** ^1^Department of Physics, IMARENAB, University of León, 24071 León, Spain; ^2^Centre for Environmental and Marine Studies (CESAM), University of Aveiro, 3810-193 Aveiro, Portugal

## Abstract

The aim of this study is to improve the estimation of the characteristic uncertainties of optic disdrometers in an attempt to calculate the efficient sampling area according to the size of the drop and to study how this influences the computation of other parameters, taking into account that the real sampling area is always smaller than the nominal area. For large raindrops (a little over 6 mm), the effective sampling area may be half the area indicated by the manufacturer. The error committed in the sampling area is propagated to all the variables depending on this surface, such as the rain intensity and the reflectivity factor. Both variables tend to underestimate the real value if the sampling area is not corrected. For example, the rainfall intensity errors may be up to 50% for large drops, those slightly larger than 6 mm. The same occurs with reflectivity values, which may be up to twice the reflectivity calculated using the uncorrected constant sampling area. The *Z*-*R* relationships appear to have little dependence on the sampling area, because both variables depend on it the same way. These results were obtained by studying one particular rain event that occurred on April 16, 2006.

## 1. Introduction

Knowledge of precipitation, and in particular of the physical characteristics of rainfall, is essential in order to construct and assess meteorological and climatic models. One of the physical parameters of raindrops is their size, associated with their volume and their fall velocity.

Various mathematical distributions have been proposed that could be adapted to drop size distributions (DSD). In [1] an exponential distribution is proposed, which is subsequently generalised by [2, 3]. Because observations confirmed that an exponential distribution overestimated the number of raindrops recorded (e.g., [4–7]), the gamma distribution was introduced. Despite the fact that other mathematical formulae have been proposed to represent drop size distributions [8, 9], the most widely used systems are the exponential and the gamma distributions, which are both still in use today (e.g., [10–14]). These distributions are also present in the efforts that have been made in the search for a normalization approach [15–20].

Raindrop size distributions reveal the microphysical mechanisms associated with the formation of precipitation [3, 21], which means that they are very useful for their assimilation in meteorological models, and for their function as an element to verify the validity of these models [22]. Also, the characteristics of the drop size distributions depend on the type of clouds (convective or stratiform) that produce them, and their stage of development [23–25]. An excellent summary of the history of the search for relationships between the microphysics of raindrops and drop size distributions can be found in [26].

As mentioned above, for many years the measurement of drop sizes has been a fundamental goal of scientists studying rainfall [27]. Progress in this field is obvious, from the earliest techniques, involving briefly exposing a sheet of absorbent paper to the rain [28] or using an uncompacted layer of flour [29, 30] to the modern equipment in use today. In [31] a method was developed consisting of taking two photographs simultaneously from two perpendicular angles; this may be considered as an intermediary stage before the development of disdrometers.

Joss and Waldvogel may be credited as having developed the first automatic disdrometer, using a microphonic sensor which transformed the vertical momentum of the impact of a drop into an electrical signal [32, 33]. Subsequently, other disdrometers were developed based on optical techniques used to measure drop sizes ([34–40] to mention some of the first ones; [41] includes a comprehensive summary). Today, all of these types of disdrometers continue to be used in scientific studies throughout the world [42–47]. In [48] we find a brief but excellent summary of research carried out with disdrometers.

Based on data from the DSD, it is possible to explore many other interesting aspects, such as the kinetic energy, momentum or reflectivity. It should be noted that the relationship between the reflectivity and the intensity of the precipitation has become a discriminating factor between convective and stratiform rain. In order to carry out some of these calculations, it is necessary to know the value of the terminal velocity of the raindrops, which may be measured or estimated. The measurements of terminal velocities made with great precision in [49] served as a basis for the empirical tests proposed in [50, 51]. However, this dependency of the terminal velocity on the size is not always taken into account [52], and this is a clear source of error when making the calculations of the derived parameters.

The fact that each drop is precipitated at a different terminal velocity means that the sampling volume of the disdrometer depends on the size of the drop considered [53]. As a result, it is easy to verify that for example, the *Z*-*R* relationships depend on the velocity of the drops [54]. However, these relationships also vary depending on the type of instrument used to take the measurements [55]. This makes it necessary to take uncertainty into account when making calculations with disdrometers, as in scientific results no numeric data makes sense unless it is accompanied by its corresponding uncertainty.

In [56] the main sources of error that affect measurements made with disdrometers are described. However, one possible error is not included: the error due to the fact that the area (and not only the volume) of the sample from the disdrometer may vary with the drop size.

In this study, we will attempt to establish the uncertainty in the sampling area of an optical disdrometer, and how this is propagated to the calculations for precipitation, as has been done for other equipment for measuring rainfall [57] or other hydrometeors [58]. More specifically, the aim of this paper is to progress in the estimation of the characteristic uncertainties of the optical disdrometer, as [59] did for the Joss-Waldvogel disdrometer, [60] for the GBPP-100 probe, or [61] for several types of disdrometers. In the case of optical disdrometers, the measurement process mainly consists of the interruption or obscuration of a laser beam when raindrops cross this beam. No problems arise when the raindrop falls perfectly within the sampling area. However, on the edges the error may be considerable and will depend on the geometric characteristics of the laser beam and on the drop size. In this paper we will attempt to quantify the sampling area and study how this influences the computation of other parameters.

In Section 2 we provide basic information on the disdrometer used. The sampling area for each drop size is calculated, and we determine the error that would have occurred if a sampling area independent of the drop size had been used. In Section 3 we describe how this error is propagated when other variables that depend on the sampling area are calculated.  Section 5 contains the conclusions and is followed by the acknowledgements and list of bibliographic references included in the text.

## 2. Disdrometer Sampling Area

**Figure 1:**
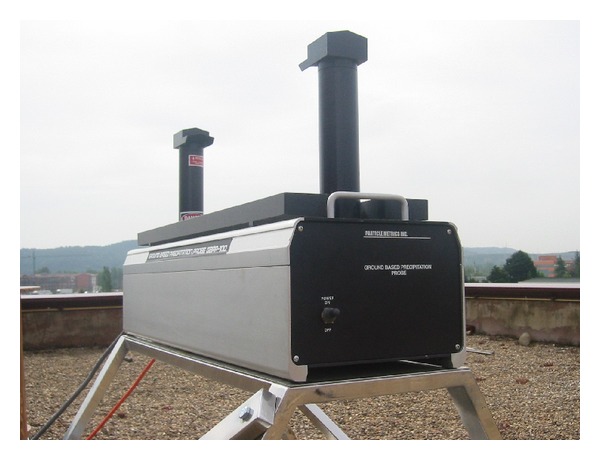
Ground-Based Precipitation Probe (PMI Model GBPP-100) installed at the University of León.

From 2003, the University of León, Spain, has carried out campaigns to gather data using an optical disdrometer during the winter. The measurement equipment considered (Figure 1) is the Ground Based Precipitation Probe (PMI Model GBPP-100). Two metres from the GBPP, at the same height over the ground, a weather station has been installed which, amongst other variables, measures wind speed and direction. It is important to know the wind speed because it may affect the reliability of the measurements taken by the GBPP; in fact, the manufacturer recommends discarding rainfall data if it is accompanied by gusts of wind stronger than 10 m/s. In our data-gathering campaigns, we have only taken into account rainfall episodes in which the wind speed did not exceed 5 m/s.

**Figure 2:**
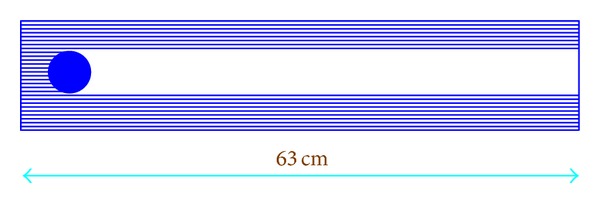
Illustration showing the measurement system of the GBPP-100, representing a laser beam intercepted by a raindrop (illustration not to scale).

The measurement system used by the GBPP is the following: the device emits a helium and neon laser beam with 64 rays (Figure 2) with a separation of 0.2 mm, and a receiver positioned 63 cm from the emitter detects how many rays are intercepted by a body (a raindrop or other object) that crosses the sampling area of 63 × 1.26 cm^2^. The number of rays intercepted corresponds to the channel in which the drop is included. 

In other words, the GBPP measures the spectrum of drop sizes from 0.2 mm, in 63 channels. The channels correspond to a given precipitation size of between 0.2 and 12.4 mm. Another channel is used to include the drops that intersect either of the two rays on the edge of the beam. In this case, the drop size is unknown.

**Figure 3:**
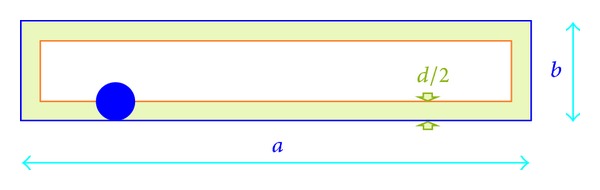
Geometry of the effective sampling area for a certain raindrop size *d*, based on the nominal sampling area.

If the nominal sampling area (shown in Figure 3) is a rectangle with dimensions *a* × *b* and we are measuring a raindrop with a diameter *d*, only raindrops centred in a rectangle with an area of (*a* − *d*)(*b* − *d*) = *ab* + *d* ^2^ − *d*(*a* + *b*) will be counted. As a result, if we suppose that the sampling area is *ab* then we are committing an error of *d* ^2^ − *d*(*a* + *b*).

It would perhaps be of interest to try and quantify this error for the case that concerns us. Firstly, it is observed that as *d* < *a* + *b*, the error we have just identified will always be negative. This means that the real sampling area is always smaller than the nominal area. In order to avoid complications with the signs, we will always refer to the absolute value of this error, namely *d*(*a* + *b*) − *d* ^2^.

For a sampling area with a value of (*a* − *d*)(*b* − *d*), supposing that *ab* is suitable means working with a quantity that is affected by a relative error:

(1) $$\begin{array}{c} \frac{d\left(a+b\right)-d^{2}}{\left(a-d\right)\left(b-d\right)}. \end{array}$$

**Figure 4:**
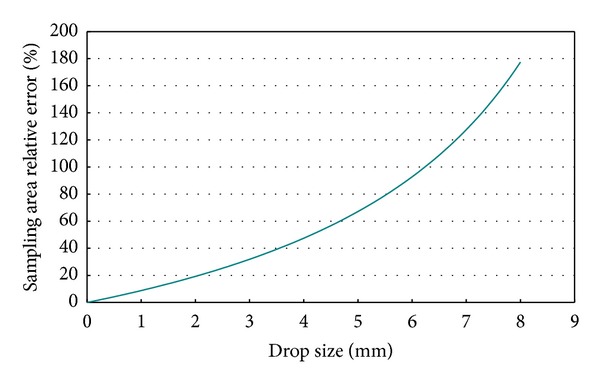
Relative error of the sampling area depending on drop size.

In this equation, considering *a* = 63 cm and *b* = 1.26 cm, the result is shown in Figure 4, indicating the relative error based on the drop size. Here we can see that for large drops (a little over 6 mm), the effective sampling area is half the area indicated by the manufacturer.

## 3. Rain Variables

The error committed in the sampling area is propagated to all of the variables that depend on this surface. Here we will refer to two of them: rain intensity or rain rate, and reflectivity factor.

**Figure 5:**
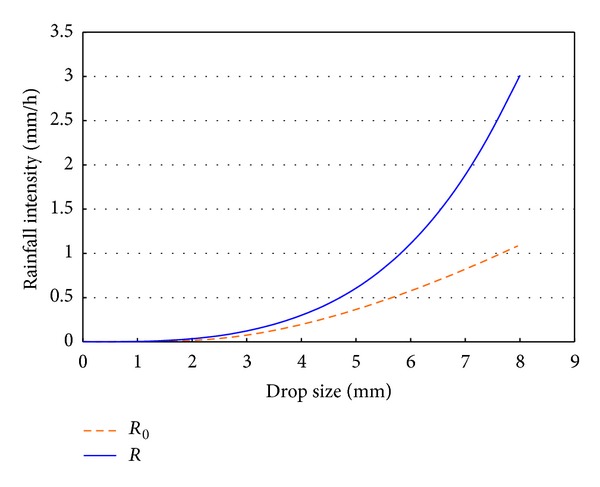
Rainfall intensities calculated with the sampling area uncorrected (*R*_0_) and corrected (*R*).

The intensity is the precipitated volume of water per unit of time and area, so it will depend on the sampling surface. It is possible to calculate the intensity *R* once the sampling surface is corrected and the intensity *R*_0_, supposing the sampling surface is constant (*ab*). On representing the two variables depending on the drop size, Figure 5 is obtained. Here we can see the precipitation intensity (*y*-axis) when a drop of a certain size (*x*-axis) falls in one minute. On producing this graph, the deformation of the drops when falling has been taken into account. This is important because the flattening of the drops means that the disdrometer always measures the largest dimension of the drop. The correction proposed in equation (1) in [51] has been used here.

Figure 5 shows that the error committed by assuming that the sampling area is constant tends to underestimate the real intensity: actually, the intensities are higher than those we calculate with a constant area. And these rainfall intensity errors may be of up to 50% for large drops, slightly more than 6 mm (larger sizes are infrequent, and drops larger than 8 mm are not registered).

Another variable that depends on the sampling area is the reflectivity factor *Z* of the rainfall, defined as in [62]. In this case, apart from the sampling area, it is necessary to know the fall velocity of the drops. As the GBPP does not measure this parameter, it is necessary to assume that it takes a certain value. A sophisticated study of the terminal velocity is described in [63], using experimental data found by other authors. In this study, we have supposed that the velocity varies with size, according to

(2) $$\begin{array}{c} v=-0.0009748D^{4}+0.05730D^{3}-0.8393D^{2}+4.712D \end{array}$$

proposed by [51], based on the measurements of [49]. 

**Figure 6:**
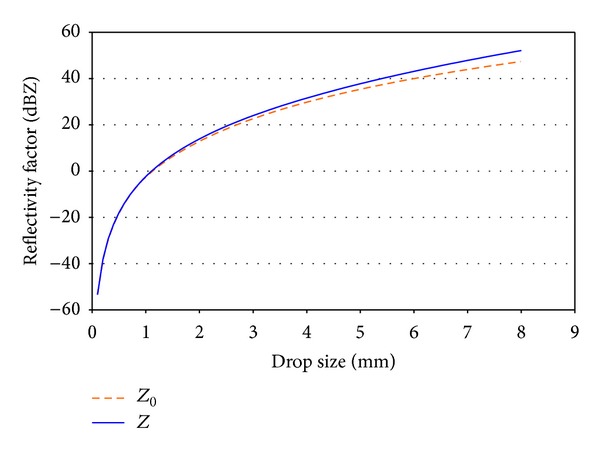
Reflectivity factors calculated with the sampling area uncorrected (*Z*_0_) and corrected (*Z*).

As in the previous case, we have used *Z* to refer to the reflectivity factor calculated with the different sampling areas, and *Z*_0_ for the reflectivity factor calculated with a constant sampling surface. Using these terms, Figure 6 shows these two reflectivity factors as a function of the drop size that falls in one minute. Once again we may see that assuming a constant sampling area results in an underestimation of the reflectivity. For example, for large drop sizes, such as 6 mm, the difference between these two reflectivities is approximately 3 dBZ. In these units the difference does not seem to be exaggerated, but we have to take into account the fact that these are logarithmic units: a difference of 3 dBZ between two reflectivities means that one is approximately twice the size of the other.

Here we have presented a calculation for monodisperse drop distributions. In a real precipitation event, if the size distribution is known (*n*_*i*_ drops of size *d*_*i*_), then Figures 5 and 6 may be used to evaluate the possible error. However, we will continue using these data for a real precipitation event.

## 4. Example: Case Study of April 16, 2006

**Figure 7:**
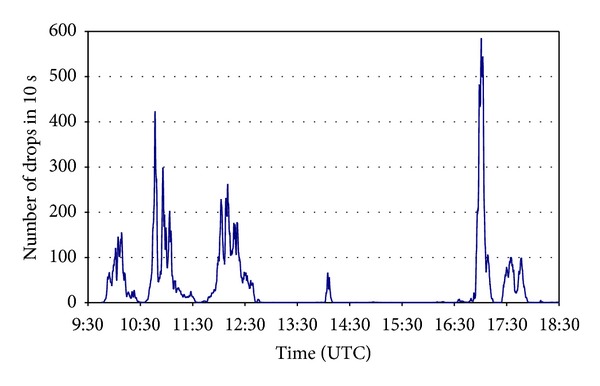
Number of drops recorded at ten-second intervals.

On April 16, 2006, atmospheric instability over the city of León led to rainfall that was not excessively intense but quite continuous, lasting between approximately from 0930 to 1800 UTC. During this interval, the GBPP detected 105,192 drops, distributed in intervals of ten seconds, as shown in Figure 7. 

**Figure 8:**
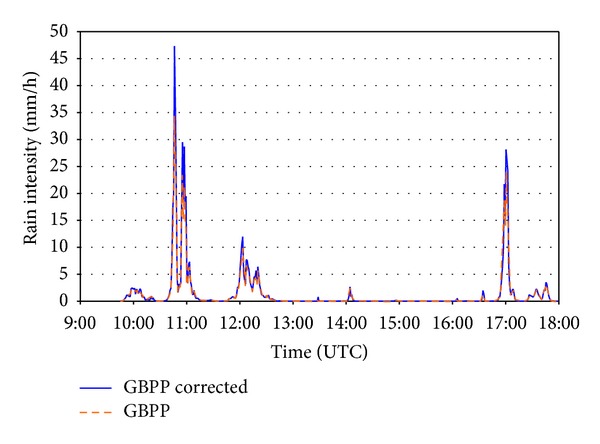
Rainfall intensities calculated with the sampling area uncorrected (*GBPP*) and corrected (*GBPP corrected*).

**Figure 9:**
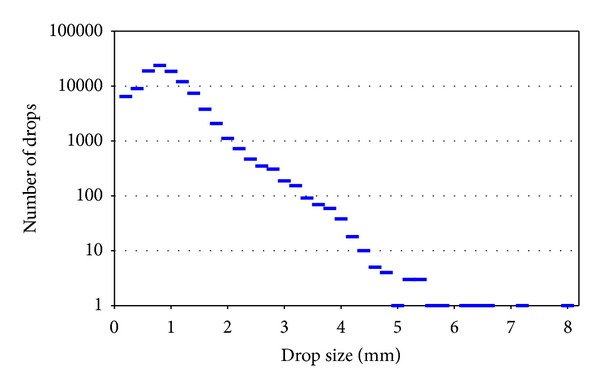
Drop size distribution of all raindrops recorded on April 16, 2006.

In order to establish comparisons with the rain gauge installed a couple of meters from the optical disdrometer, the data are shown in one-minute intervals.  Figure 8 shows the intensity of the rainfall per minute calculated with the constant sampling area and with the sampling area that varies according to the drop size. It was seen that the intensity (GBPP corrected) calculated with the variable sampling area is always higher than the other one (shown as GBPP). The fact that the intensity is not proportional to the number of drops in Figure 7 indicates that the distribution of sizes is different in each precipitation event. The data for the whole rain event recorded by the DSD are shown in Figure 9 and indicate a distribution that is closer to a gamma distribution than to the exponential distribution in [1].

**Figure 10:**
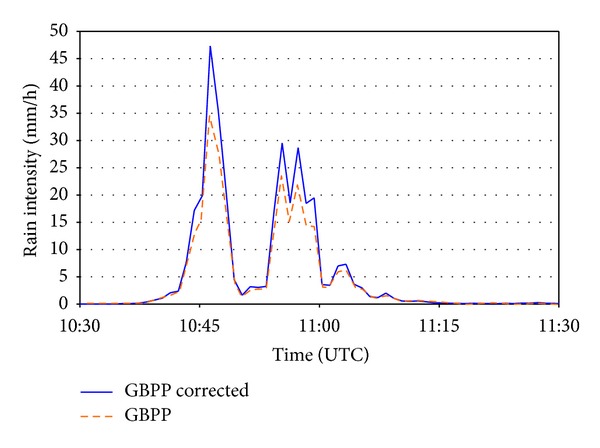
Rainfall intensities calculated with the sampling area uncorrected (*GBPP*) and corrected (*GBPP corrected*) during the most intense rainfall episode.

With the aim of focusing our example on an uninterrupted rainfall episode over a certain period of time, we decided to select one of the intervals shown in Figure 8, more precisely the one that shows the highest precipitation intensity, registered approximately between 1030 and 1130 UTC. This interval is amplified in Figure 10, showing more clearly the difference between the two intensities, especially in the precipitation peaks, perhaps because the drop sizes recorded are larger. We should not forget that the larger the drop size, the larger the correction that needs to be introduced in the sampling area.

**Figure 11:**
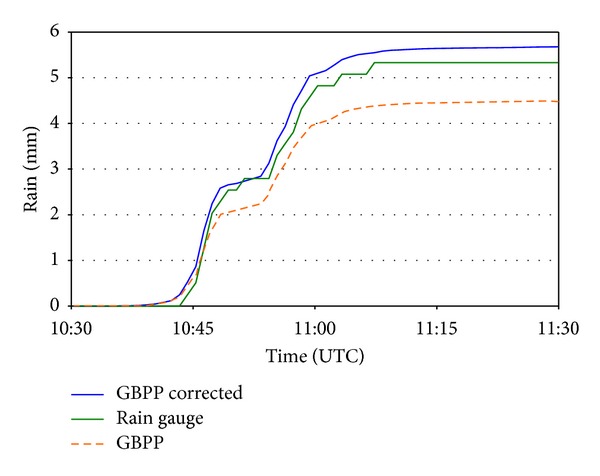
Comparison between the total rainfall recorded by the GBPP and by the rain gauge during the most intense rainfall episode.

The scope of the correction proposed in this study would be quite reduced, and perhaps reserved for theoretical use, if we did not compare it with other types of measurements that avail its transcendence. Together with the GBPP, the meteorological station contained a tipping bucket rain gauge, so we have been able to compare the precipitation recorded. For the episode we have studied, Figure 11 shows the accumulated precipitation as measured by the rain gauge and as calculated according to the data from the GBPP, with and without correction of the sampling area.

Of course we must bear in mind that the differences between the values measured by disdrometer and by rain gauges are due to a number of facts other than the sampling area, such as the discretization of diameter [64, 65], minimum detectable drop size, and others. Figure 11 corroborates that the precipitation calculated with the correction of the sampling area is higher than with the nominal sampling area. However, the most interesting aspect is that the values provided by the rain gauge are, generally, closer to those calculated with the corrected sampling area. We therefore argue that, although disdrometers generally tend to measure lower rainfall values than rain gauges, a correction of the sampling area may reduce these differences.

**Figure 12:**
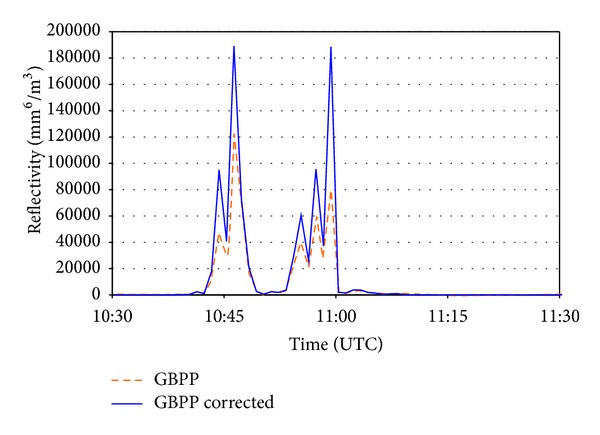
Reflectivity factors calculated with the sampling area uncorrected (*GBPP*) and corrected (*GBPP corrected*) during the most intense rainfall episode.

The other variable studied in the previous epigraph is reflectivity, *Z*.  Figure 12 shows the reflectivity values calculated as previously indicated. Once again, on reducing the sampling area the reflectivity increases, and the values found are twice those made using the nominal sampling area. This difference, which is evident when *Z* is represented on a linear scale, is eclipsed if the scale is logarithmic (e.g., when it is represented in dBZ units). It may be seen that in some minutes, the difference is as much as around 3 dBZ, which represents a ratio equal to 2 in the reflectivity.

**Figure 13:**
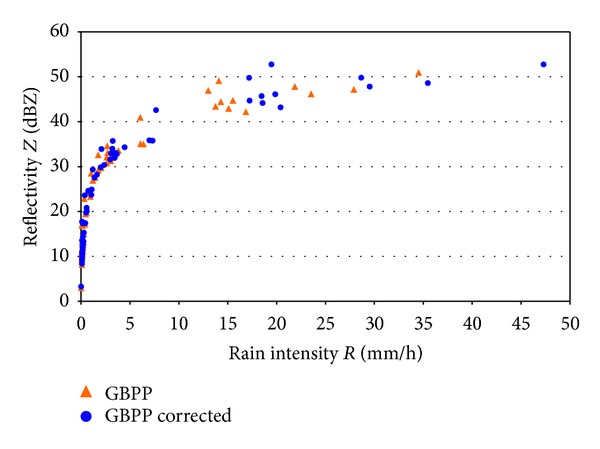
*Z*-*R* relationship calculated with the sampling area uncorrected (*GBPP*) and corrected (*GBPP corrected*).

Finally, we will deal with the *Z*-*R* relationships, which are of interest in order to know the type of precipitation.  Figure 13 shows the distribution of the reflectivity based on the precipitation intensity during the minutes of precipitation we have just studied. The curves of best fit are not shown, as they are both superimposed over each other. In fact, their equations are *Z* = 376*R* ^1.51^ for the corrected data, and *Z* = 382*R* ^1.50^ for the data calculated with the nominal sampling area. The correlation is also similar (*r* ^2^ = 0.968 and 0.969, resp.).

So why are there no differences in the *Z*-*R* relationships, when both *Z* and *R* individually had a different behavior, which was strongly dependent on the sampling area? The answer is simply that the two variables depend on the sampling area in the same way: both variables increase with the proposed correction, so this relationship seems to have little dependence on the sampling area.

Of course, it will be necessary to have a more extensive database in order to generalize this result, including not only more rainfall episodes with different characteristics, but also rainfall data from other locations, due to the strongly regional nature of the *Z*-*R* relationship [66–68].

To conclude, it seems clear that any other variable we calculate (energy, linear momentum, size spectrum, etc.) which is dependent on the sampling area will have to be corrected according to the guidelines indicated in this paper. In this case, comparing the databases with those for disdrometers with transfer of momentum [32] in order to corroborate the corrections of the sampling area would be a good research line.

## 5. Conclusions

The main conclusions of this study are the following.

- When calculating the variables based on the data from the disdrometer it is necessary to take into account the real sampling area (variable for each drop size): it is not enough to take a constant area, which may be the one indicated by the manufacturer. Otherwise, this leads to major errors in the calculations of the derived variables.
- One of the most important errors is the one found in calculating the rainfall intensity *R*, which may be as much as 50% of the rainfall for the largest drop sizes. For this reason, once we know to what degree of accuracy we have to know *R* and the size of the raindrops recorded, we will be able to determine if we need to introduce the correction of the sampling area.
- Another variable that may also be affected is the reflectivity factor *Z*, which when calculated using the variable sampling area may be up to twice the reflectivity calculated using the uncorrected constant sampling area.
- In contrast, the *Z*-*R* relationship seems to have little dependence on the sampling area, because the errors of *Z* and *R* tend to be compensated.
- With actual rain records, it was observed that the fact that the intensity is not proportional to the number of raindrops indicates that the distribution of sizes is different in each precipitation episode, even on the same day. The global data for the drop size distribution indicate that it is more similar to a gamma distribution than to an exponential one.
- Although disdrometers generally do not provide exactly the same rainfall values as rain gauges, a correction of the sampling area could reduce these differences.

In conclusion, the nominal area of the sampling should not be considered as final, without previously calculating the possible error we may introduce into the calculations.
